# Non-destructive detection of kiwifruit soluble solid content based on hyperspectral and fluorescence spectral imaging

**DOI:** 10.3389/fpls.2022.1075929

**Published:** 2023-01-18

**Authors:** Lijia Xu, Yanjun Chen, Xiaohui Wang, Heng Chen, Zuoliang Tang, Xiaoshi Shi, Xinyuan Chen, Yuchao Wang, Zhilang Kang, Zhiyong Zou, Peng Huang, Yong He, Ning Yang, Yongpeng Zhao

**Affiliations:** ^1^ College of mechanical and electrical engineering, Sichuan Agriculture University, Ya’an, China; ^2^ College of Engineering, Chinese University of Hong Kong, Hong Kong, Hong Kong SAR, China; ^3^ College of Biosystems Engineering and Food Science, Zhejiang University, Hangzhou, China; ^4^ School of Electical and Information Engineering, Jiangsu University, Zhenjiang, China

**Keywords:** hyperspectral, fluorescence spectral, non-destructive detection, kiwifruit, ssc

## Abstract

The soluble solid content (SSC) is one of the important parameters depicting the quality, maturity and taste of fruits. This study explored hyperspectral imaging (HSI) and fluorescence spectral imaging (FSI) techniques, as well as suitable chemometric techniques to predict the SSC in kiwifruit. 90 kiwifruit samples were divided into 70 calibration sets and 20 prediction sets. The hyperspectral images of samples in the spectral range of 387 nm~1034 nm and the fluorescence spectral images in the spectral range of 400 nm~1000 nm were collected, and their regions of interest were extracted. Six spectral pre-processing techniques were used to pre-process the two spectral data, and the best pre-processing method was selected after comparing it with the predicted results. Then, five primary and three secondary feature extraction algorithms were used to extract feature variables from the pre-processed spectral data. Subsequently, three regression prediction models, i.e., the extreme learning machines (ELM), the partial least squares regression (PLSR) and the particle swarm optimization - least square support vector machine (PSO-LSSVM), were established. The prediction results were analyzed and compared further. MASS-Boss-ELM, based on fluorescence spectral imaging technique, exhibited the best prediction performance for the kiwifruit SSC, with the ${\text{R}}_{p}^{2}$ , ${\text{R}}_{c}^{2}$ and RPD of 0.8894, 0.9429 and 2.88, respectively. MASS-Boss-PLSR based on the hyperspectral imaging technique showed a slightly lower prediction performance, with the ${\text{R}}_{p}^{2}$ , ${\text{R}}_{c}^{2}$ , and RPD of 0.8717, 0.8747, and 2.89, respectively. The outcome presents that the two spectral imaging techniques are suitable for the non-destructive prediction of fruit quality. Among them, the FSI technology illustrates better prediction, providing technical support for the non-destructive detection of intrinsic fruit quality.

## 1 Introduction

People love kiwifruit for its sweet and sour taste and rich nutritional value. Sugar is important in judging kiwifruit’s quality, affecting its taste. About 81% of kiwifruit’s solid soluble content (SSC) is sugar, so SSC is usually used to evaluate its sugar content. The traditional SSC detection methods use refractometer and other instruments, which are cumbersome to operate and also destroy the physical integrity of the detected object, and cannot achieve rapid detection. Therefore, realizing the non-destructive detection of kiwifruit SSC is of great practical importance.

Hyperspectral imaging (HSI) and fluorescence spectral imaging (FSI) technologies combine image and spectral information, which can quickly detect the quality parameters of the measured object without damage. In recent years, HSI technology has developed rapidly in the non-destructive detection of the intrinsic parameters of fruits, such as SSC, pH, hardness, etc. Pham et al. (Pham and Liou, 2022) used HSI to achieve online detection of jujube surface defects. They used principal component analysis (PCA) to extract feature variables from hyperspectral data in a spectral range of 468~950 nm to establish ANN and SVM models, illustrating accuracy rates of 95% and 94.6%, respectively. Li et al. (Li et al., 2022) used short-wave infrared HSI technology to predict the SSC in dried Hami jujube and established the FS-CNN model, where ${\text{R}}_{p}^{2}$ and RPD were 0.857 and 2.648, respectively. Gao et al. (Gao and Xu, 2022) predicted the SSC of red globe grape by combining HSI imaging technology with the PLSR model. They obtained the correlation coefficients of the calibration and prediction sets of 0.9775 and 0.9762, respectively.

FSI technology utilizes the fluorescence of different intensities emitted by excited molecules or atoms when certain substances are excited after being irradiated by light of specific wavelengths. Compared with HSI technology, FSI technology was applied later but achieved good progress in recent years. For example, Kim et al. (Kim et al., 2022) used FSI technology to detect aflatoxin in corn under 365 nm ultraviolet excitation rapidly, and the detection accuracy of the quadratic support vector machine (QSVM) reached 95.7%. Zhou et al. (Zhou et al., 2022) used FSI technology to detect the heavy metal lead in lettuce leaves, where a fluorescent filter of 475 nm was used to collect the fluorescence spectrum image in the spectral range of 480.46 nm~1001.61 nm, and ${\text{R}}_{c}^{2}$ , ${\text{R}}_{p}^{2}$ and RPD of the best prediction method (i.e., WT-MS-SAE-SVR) were 0.9802, 0.9467, and 3.273, respectively. Kang et al. (Kang et al., 2022) used FSI technology to detect the dry matter content of mango, and ${\text{R}}_{c}^{2}$ , ${\text{R}}_{p}^{2}$ , RMSEC and RMSEP of the best prediction method (i.e., CARS-RF-SPA-BPNN) were 0.9710, 0.9658, 0.1418 and 0.1526, respectively.

Although FSI technology has been widely used to detect agricultural products, most current studies are extended to detect mold, and less is applied to detect the intrinsic quality of agricultural products. In this study, the feasibility of FSI technology to predict kiwifruit SSC was examined, and the outcome was compared with HSI technology, where the feature extraction method was designed to establish a prediction model and the effects of two different imaging technologies on the performance of the prediction model were analyzed from the experimental results. Also, various regression prediction models were compared, and the performance differences between the two detection techniques led to the best method for detecting kiwifruit SSC.

## 2 Materials and methods

### 2.1 Materials

90 samples of “Hongyang” kiwifruit with intact skin were selected from a kiwifruit base in Ya’an City, Sichuan Province. After the sample’s surface was cleaned with water, they were sequentially numbered and left at room temperature (25 ± 1 °C) for 24 h, and their hyperspectral and fluorescence spectral images were collected. After collecting the two spectral images of the samples, their SSC physicochemical values were determined immediately. According to the SSC measurement method of “NY/T 2637-2014”, the samples were washed and peeled around their equators, then the pulp was removed, and the juice was pressed. The fruit juice was introduced into the detection tank of the handheld glucose salinity refractor (i.e., YSK-107) with a resolution of 0.1% Brix, and the data were recorded as the SSC physicochemical values after the display data were stable. In order to reduce the operation error, each sample was measured twice, and the average value was taken as the SSC physicochemical value of the sample, with the unit of “Brix”.

### 2.2 Acquisition equipment

**Figure 1:**
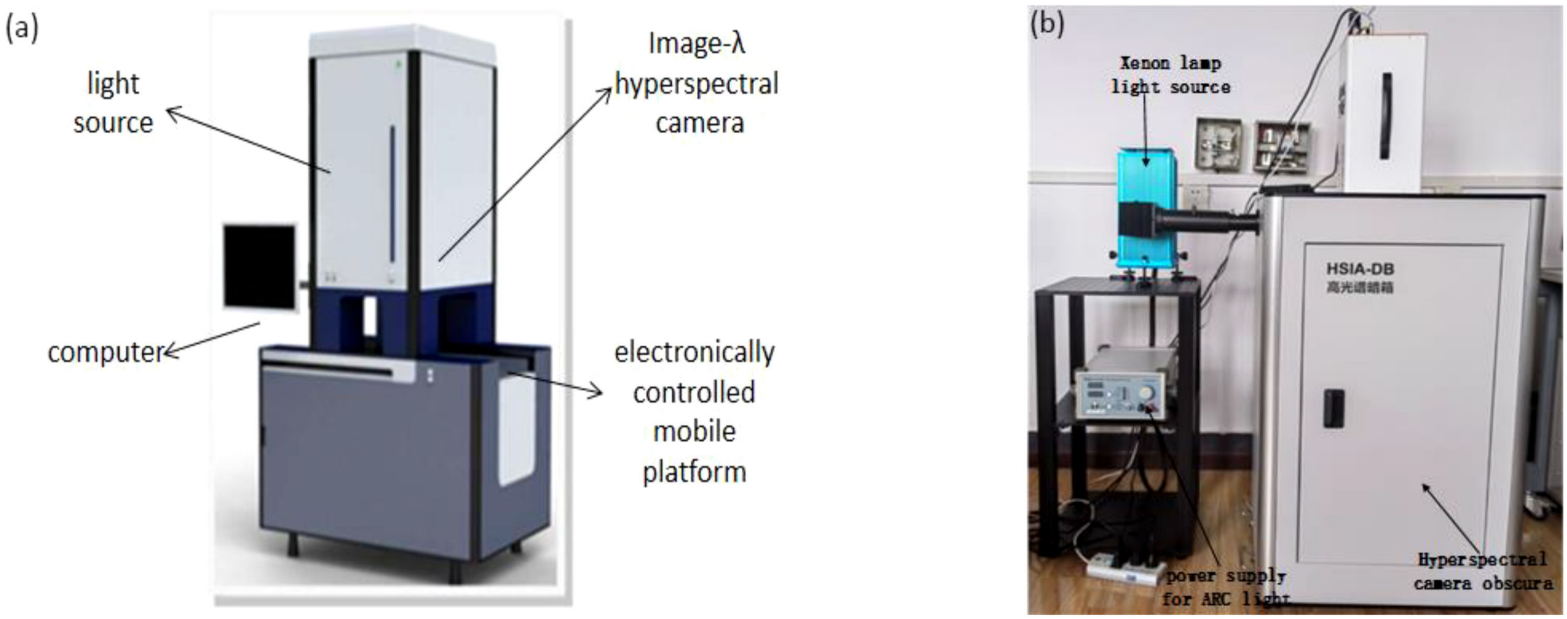
The overall equipment structure: **(A)** Gaia hyperspectral sorter; **(B)** Gaia fluorescence spectral detection system.

Hyperspectral images of the kiwifruit samples were collected by Gaia sorter “Gaia” hyperspectral sorter in a spectral range of 387~1034 nm. The sorter mainly includes two groups of 4 LSTS-200 bromine tungsten lamps with a uniform light source, Image-Λ “spectral image” series CCD camera, an electronically controlled mobile platform and a computer with hyperspectral data acquisition software (SpaceView) powered by AC220V. The pixel and pixel size of the spectral camera are 1344 × 1024 and 6.45 × 6.45 μm, respectively. The overall structure of the Gaia sorter is shown in **Figure 1A**.

The GaiaFluo series fluorescence spectral detection system was utilized to collect the fluorescence images of kiwifruit samples. In the system, the camera is Gaiafluo-VN-HR, the spectrometer is a transmission grating (PGP) structure, the spectral range and resolution are 400-1000 nm and 2.8 nm, respectively, and the detector is the SCMOS with a pixel size of 6.5 μm. The system also includes an 80 × 80 × 100 cm Obscura and a 30 × 30 × 40 cm platform. In addition, it contains four 50 W reflective light sources, a 150 W xenon lamp light source, various excitation filters, fluorescent filters, and a computer equipped with spectrum acquisition software (spaceview). The overall structure of the fluorescence spectral detection system is shown in **Figure 1B**.

### 2.3 Spectral image acquisition

The HSI system was first warmed up for more than 30 min before the hyperspectral images of the samples were acquired and corrected in black and white after stabilizing the voltage. During acquisition, the sample platform was 170 mm away from the lens, the exposure time of the spectroscopic camera was 13.5 ms, the advancing distance of the electronically controlled platform was 110 mm, and the advancing and retracting speeds were 4.6 mm/s and 50 mm/s, respectively. Similarly, the FSI system was prewarmed for about 30 min, and suitable excitation and fluorescence filters were selected after stabilizing the voltage. A xenon lamp was selected as the excitation light source.

After the combination of different filters was tested, the excitation filter with a central wavelength of 390 nm and a bandwidth of 40 nm and the fluorescence filter with a central wavelength of 495 nm were finally selected. The system parameters were set as follows: the camera moving speed was 0.13 mm/s, the exposure time was 800 ms, and the distance between the spectral camera lens and the measured object was about 70 cm.

**Figure 2:**
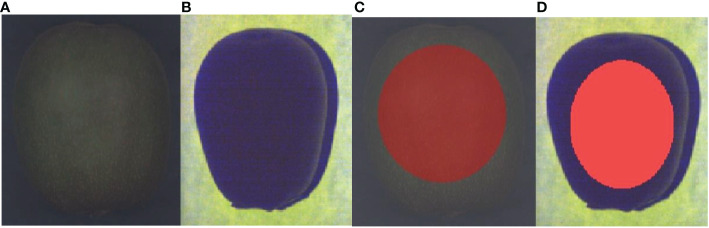
Spectral image of a sample: **(A)** raw hyperspectral image; **(B)** raw fluorescence spectral image; **(C)** ROI of the raw hyperspectral image; **(D)** ROI of the raw fluorescence spectral image.

The HSI and FSI spectral images of the samples and their region of interest (ROI) are shown in **Figures 2A–D**, respectively. During the acquisition process of hyperspectral images, SpecView software was employed to perform black-and-white calibration on the hyperspectral images to reduce the interference of environmental factors, and ENVI 5.3 software was utilized to extract the ROI. The average spectrum in the ROI was taken as the raw spectral value of the samples.

### 2.4 Methods

#### 2.4.1 Spectral pre-processing methods

Collecting spectral image data is easily affected by the differences between samples, environmental noise, and baseline drift during detection. In order to reduce these interferences, selecting appropriate pre-processing methods for the raw spectral images is necessary. Among the common pre-processing methods, the standard normal variant transform (SNV) (Dong et al., 2022, Liu et al., 2022) eliminates the error caused by different scattering levels between samples. The detrend correction (DT) (Ai et al., 2022) reduces the influence of external noise on the spectral curve by subtracting the trend-fitting line of the noise. The Savitzky-Golay (SG) convolution smoothing (Ren et al., 2021) reduces the noise by smoothing the spectral data within the window. The Gaussian window smoothing (GWS), boxing smoothing (BS) and exponential smoothing (ES) methods can reduce the noise in different smoothing ways.

#### 2.4.2 Feature extraction methods

The pre-processed spectral data exhibited a multicollinearity problem, so it was necessary to find the feature variables beneficial to the prediction results and eliminate the invalid variables. In this study, the Bootstrapping soft shrinkage (Boss) algorithm (Deng et al., 2016; Ouyang et al., 2021), the competitive adaptive reweighted sampling (CARS) algorithm (Zhang et al., 2019; Shicheng et al., 2021), the iteratively variable subset optimization (IVSO) algorithm (Sun et al., 2021), the Interval Variable Iterative Space Shrinkage Approach (IVISSA) (Cheng et al., 2020; Hao et al., 2022) and the Model adaptive space shrinkage (MASS) (Wen et al., 2016) methods were used to extract the spectral data.

#### 2.4.3 The modeling methods

Extreme learning machines (ELM) is a single-hidden layer feedforward neural network with fast training speed and strong generalization ability. It is widely used in various classification and regression scenarios (Jiang et al., 2018; Cheng et al., 2022). The partial least squares regression (PLSR) model combines principal components analysis (PCA) with maximum correlation analysis to fit the distribution of random variables into linear equations. It is widely used in mathematics, statistics, and finance (Guo et al., 2021; Ma et al., 2021). Least square support vector machine (LSSVM) replaces the complex secondary optimization problem in the traditional SVM by solving primary linear equations, simplifying the model and improving its operation speed (Feng et al., 2018; Zhang et al., 2020).

#### 2.4.4 The evaluation indicators

Five indicators, namely the coefficient of determination of the calibration set ( $R_{c}^{2}$ ), the root mean square error of the calibration set (RMSEC), the coefficient of determination of the prediction set ( $R_{p}^{2}$ ), the root mean square error of the prediction set (RMSEP), and the residual prediction deviation (RPD) were selected to evaluate the prediction capabilities of the developed models (Sharma et al., 2022). These evaluation indexes were calculated using the following Eqs. (1)-(3).


(1) $$R^{2\;}=\;1-\frac{\sum_{i=1}^{n}{\left(y_{i}-f_{i}\right)}^{2}}{\sum_{i=1}^{n}{\left(y_{i}-\bar{y}\right)}^{2}}\in [0,\;1]$$

(2) $$\text{RMSE=}\sqrt{\frac{\sum_{i=1}^{\text{n}}{\left({\text{f}}_{\text{i}}{\text{-y}}_{\text{i}}\right)}^{2}}{\text{n}}}$$

(3) $$\text{RPD=}\frac{{\text{Std}}_{\text{p}}}{\text{RMSEP}}$$

where R^2^ represents the correlation between the predicted and actual values, and the closer R^2^ is to 1, the better the predictive stability and the fit of the model. RMSE represents the difference between the predicted and actual values, and a smaller RMSE indicates better model prediction performance. RPD is the ratio of the sample’s standard deviation, and its root means square error (Saeys et al., 2005). RPD< 1.4 indicates a poor model prediction, 1.4 ≤ RPD ≤ 2 indicates an average model prediction and RPD ≥ 2 indicates a good model prediction.

#### 2.4.5 The optimization method

The particle swarm optimization (PSO) algorithm was originally proposed by Eberhart and Kennedy in 1995 and used commonly to solve optimization problems (Bhandari et al., 2015; Bonah et al., 2020). Its principle indicates that the position of each particle corresponds to the optimal vector of the problem to be solved, and a population X of m particles in a D-dimensional space is set. The position *X_i_* and the moving speed V_i_ of the *i*th particle in the population corresponds to (*X*_*i*1_,*X*_*i*2_,*X*_*i*3_,…*X*_*iD*_) and (V_i1_,V_i2_,V_i3_,…V_iD_) , respectively, and P_ibest_ is (P_i1_,P_i2_,P_i3_,…P_iD_)  , representing the optimal position sought by the individual particles. At this time, the global optimal position of the whole population is G_best_, which is (P_g1_,P_g2_,P_g3_,…P_gD_) . Each particle continuously updates P_best_ and G_best_ through a given fitness function until the optimal solution is found or the number of iterations is reached. The velocity and position of the *d*th-dimension of the *i*th particle are updated as follows (Eqs (4) and (5)).


(4) $${\text{V}}_{\text{id}}^{k+1}{\text{=wV}}_{\text{id}}^{\text{k}}{\text{+c}}_{1}{\text{r}}_{1}\left({\text{P}}_{\text{id}}^{\text{k}}{\text{-X}}_{\text{id}}^{\text{k}}\right){\text{+c}}_{2}{\text{r}}_{2}\left({\text{P}}_{\text{gd}}^{\text{k}}{\text{-X}}_{\text{id}}^{\text{k}}\right)$$

(5) $${\text{X}}_{\text{id}}^{k+1}{\text{=X}}_{\text{id}}^{\text{k}}{\text{+X}}_{\text{id}}^{k+1}$$

where c_1_ and c_2_ are learning factors which adjust the maximum step size of learning, r_1_ and r_2_ are random numbers in the range of 0~1, and w is the inertia weight that adjusts the searchability of the solution space. This study used the PSO algorithm to optimize the LSSVM model parameters.

## 3 Results and discussion

### 3.1 Original spectral data

**Figure 3:**
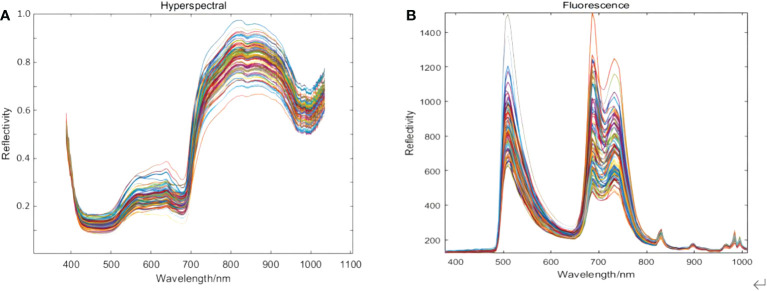
Spectral data of kiwifruit acquired by using **(A)** hyperspectral imaging and **(B)** fluorescence imaging.

The raw spectral curves of the 90 kiwifruit samples are shown in **Figure 3**. **Figure 3A** is the original hyperspectral data in a wavelength range of 387.15 nm~1034.99 nm with 256 spectral bands. **Figure 3B** is the original fluorescence spectral data in a wavelength range of 376.80 nm~1011.05 nm with 125 spectral bands.

It can be seen from **Figure 3A** that the bands at the beginning and the end of the original hyperspectral image data are significantly affected by noise. The spectral range of 420 nm~1000 nm was selected as the useful wavelength for the original hyperspectral image, with a total of 229 spectral bands. From **Figure 3A**, the troughs at 450 nm and 670 nm could be due to chlorophyll and other pigments in the cell wall. In comparison, the trough absorption peak at 980 nm is attributed to the tertiary and secondary frequencies of the C-H and O-H bonds in kiwifruit SSC (Chu, 2016). The first and last bands of the original fluorescence spectral images were also affected by noise, so the spectral range of 400~900 nm was selected as the effective wavelength of the original fluorescence spectral images, with a total of 102 spectral bands. From **Figure 3B**, after using the excitation filters with a central wavelength of 390 nm and 495 nm, obvious peaks appear near 510 nm, 690 nm, and 740 nm.

### 3.2 Sample division

Dividing samples are beneficial to the stability and accuracy of the model prediction. Kennard Stone (KS) (Wei et al., 2020; Huang et al., 2021) algorithm was applied to divide 90 samples into a training set of 60 samples and a prediction set of 30 samples in a ratio of 2:1. The SSC values were collected by a handheld YSK-107 Brix salinity refractometer. The statistical results of the training and prediction sets of HSI and FSI are listed in **Table 1**.

**Table 1:** Statistical results of training and prediction data sets of SSC (unit:/Brix).

	Sample set	Number of samples	Minimum value	Maximum value	Average value	Standard deviation
Hyperspectral sample set division	Training set	60	6.50	14.90	10.97	1.79
	Prediction set	30	8.70	15.35	11.41	1.44
Fluorescence spectral sample set division	Training set	60	6.50	15.35	11.04	1.79
	Prediction set	30	8.70	14.90	11.27	1.46

From **Table 1**, the ranges of each statistical parameter for the SSC values of the training set and prediction set samples corresponding to the HSI data are 6.50~14.9 and 8.70~15.35, respectively, and the standard deviations of the two samples are 1.79 and 1.44, respectively. Although the data range of the prediction set exceeds the training set, only occasional individual data at the front and back ends of the data exist. By comparing the standard deviations, the data of the prediction set are more concentrated, conforming to the principle of independent and identical distribution, indicating that the distribution of the two is relatively consistent. The statistical parameters of the SSC values of the training set and the prediction set corresponding to the FSI data ranged from 6.50 to 15.35 and 8.70 to 14.90, respectively. The above results illustrate that the sample division is reasonable and representative.

### 3.3 Spectral pre-processing

The raw effective spectral image data were pre-processed by the above six methods, and the prediction results of each pre-processing method were compared through the PLSR model, from which the optimal pre-processing method was selected. The prediction results of PLSR are listed in **Table 2**. The number of latent variables (*lvs*) in **Table 2** was determined by the cross-sectional analysis. 1 to *n* potential variables were used to establish the model and the number of *lvs* with the best prediction was selected.

**Table 2:** The prediction results of PLSR based on different pre-processing methods.

	Methods	lvs	${\text{R}}_{c}^{2}$	*RMSEC*	${\text{R}}_{p}^{2}$	*RMSEP*	*RPD*
Hyperspectral	H-Raw data	10	0.8095	0.7739	0.6795	0.7994	1.85
	SNV	14	0.9489	0.3610	0.4710	1.2047	1.10
	DT	14	0.9315	0.4269	0.6719	1.0052	1.19
	SG	12	0.8207	0.7507	0.7439	0.7146	2.10
	GWS	13	0.8520	0.6820	0.7334	0.7291	2.10
	BS	13	**0.8416**	**0.7055**	**0.7629**	**0.6876**	**2.21**
	ES	13	0.8481	0.6909	0.7490	0.7075	2.14
Fluorescence Spectral	F-Raw data	15	0.8689	0.6436	0.6143	0.8894	1.41
	SNV	12	0.7454	0.8894	0.4248	1.1170	1.03
	DT	12	0.7829	0.8367	0.4350	1.0423	0.99
	SG	16	**0.9021**	**0.5562**	**0.6396**	**0.8598**	**1.67**
	GWS	19	0.8764	0.6188	0.5977	0.9401	1.45
	BS	17	0.8889	0.5926	0.6030	0.9023	1.60
	ES	19	0.8618	0.6607	0.6267	0.8750	1.53

The bold values represent the best performer in each table.

During pre-processing of the hyperspectral data, the RPD values of SG, GWS, BS and ES were above 2.1 (**Table 2**), among which BS-PLSR exhibited the best prediction performance. The ${\text{R}}_{c}^{2}$ of BS-PLSR is 0.8416, which is not the optimal value, but its ${\text{R}}_{p}^{2}$ and RPD are 0.7629 and 2.21, respectively, the best values observed among all the methods. Hence BS was selected as the pre-processing method for raw hyperspectral image data. During pre-processing of the fluorescence spectral data, the RPD values of SG, BS, and ES were higher than the original fluorescence spectral data, with a value of 1.41. Among them, SG-PLSR showed the best prediction performance, and its ${\text{R}}_{c}^{2}{\text{ , R}}_{\text{p}}^{2}\text{ , and RPD}$ were 0.9021, 0.6396, and 1.67, respectively. SG was selected as the pre-processing method for raw fluorescence spectral image data.

### 3.4 Extraction of spectral feature variable

#### 3.4.1 Extraction based on boss feature variables

**Figure 4:**
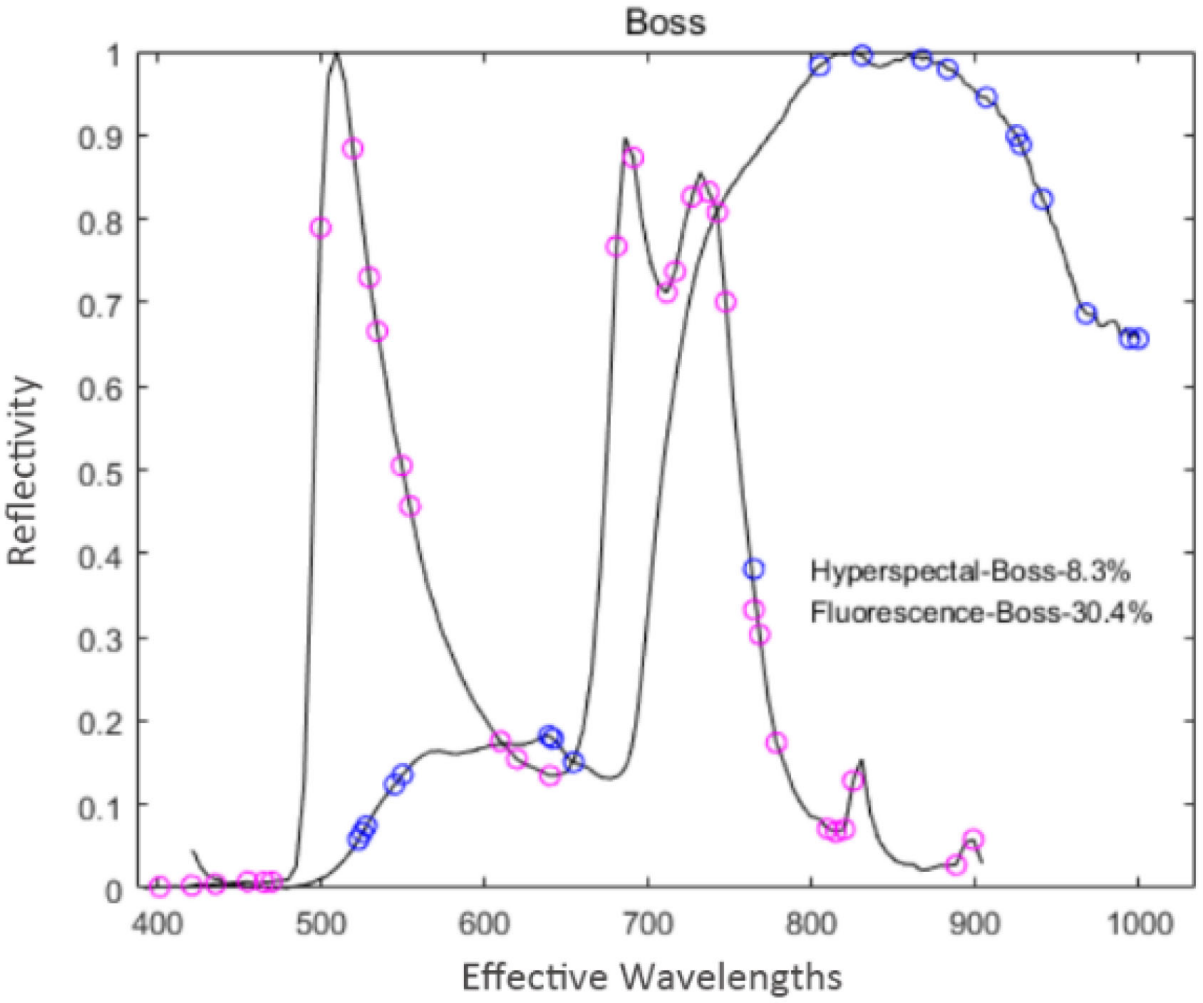
Distribution of the feature variables extracted by Boss.

Boss used WBS technology to establish a sub-model to extract feature variables randomly from the pre-processed spectral data; thus, there was certain randomness. In the experiment, Boss was repeated several times to reduce the influence of randomness. During the extraction of hyperspectral data, the number of latent variables was set to 17 through cross-validation, the cross-folding was 5 layers, and the number of sampling was 1000. Meanwhile, 19 feature variables were extracted, accounting for 8.3% of the total hyperspectral variables. Similarly, the same extraction process was performed for the fluorescence spectrum data. The number of latent variables was set to 20, other parameters were the same as above, and 31 characteristic variables were finally extracted, accounting for 30.4% of the total fluorescence spectral variables. The distribution of the feature variables extracted by Boss is shown in **Figure 4**.

As shown in **Figure 4**, the number and distribution of feature variables extracted by the Boss for the two spectral data differ. For hyperspectral data, the distribution of feature variables was mainly concentrated in the intervals of 500~650 nm and 800~1000 nm. In contrast, the feature variables were mainly concentrated in the wave peaks and troughs for the fluorescence spectral data.

#### 3.4.2 Extraction of feature variables based on CARS

**Figure 5:**
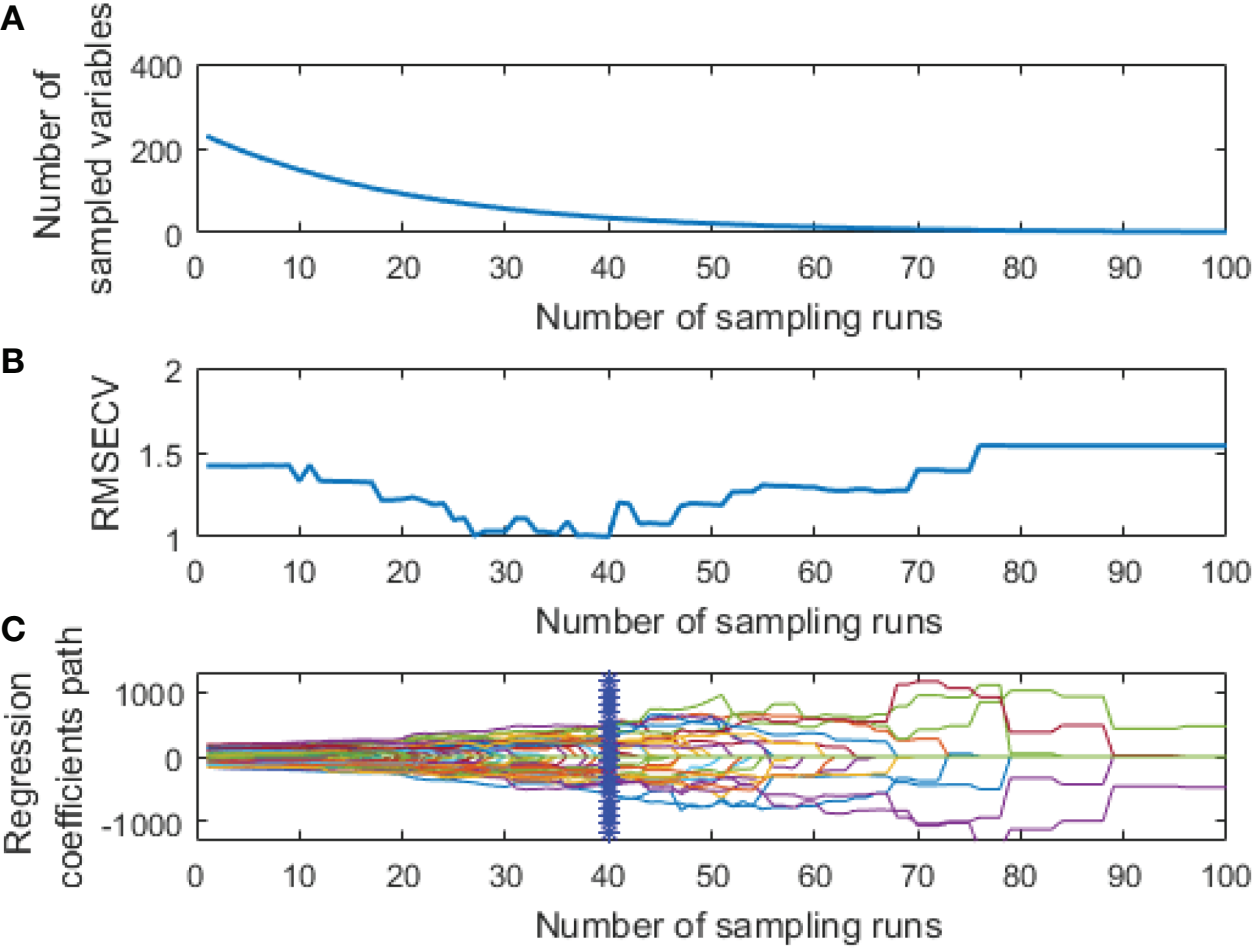
Extraction process of hyperspectral feature variables by CARS: **(A)** The number of feature variables reserved; **(B)** RMSECV; **(C)** The change of regression coefficient of each characteristic variable.

**Figure 6:**
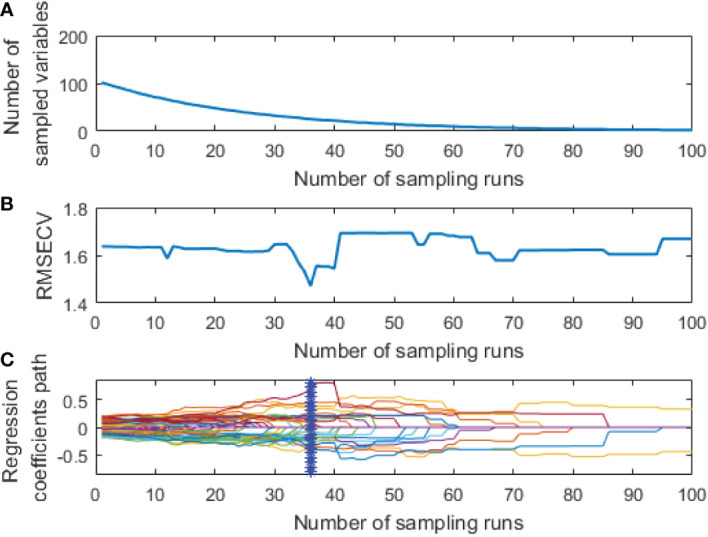
Extraction process of fluorescence spectral feature variables by CARS: **(A)** The number of feature variables reserved; **(B)** RMSECV; **(C)** The change of regression coefficient of each characteristic variable.

CARS was used to extract the feature variables from the pre-processed spectral data. The same parameters were set for both spectral data: a maximum principal component of 18, the cross-validation of 5 times, and the Monte Carlo sampling 100 times. The extraction process of two feature variables from the spectral data by CARS is shown in **Figures 5**, **6**, respectively.

As shown in **Figures 5A, B**, the number of retained feature variables showed a fast and then slow continuous decreasing trend with the increase of sampling times, while the *RMSECV* value showed a decreasing and then an increasing trend. This could be due to the elimination of many redundant variables at the initial extraction stage. However, the excessive deletion of variables at the later extraction stage led to a decline in the model’s prediction performance.

**Figure 7:**
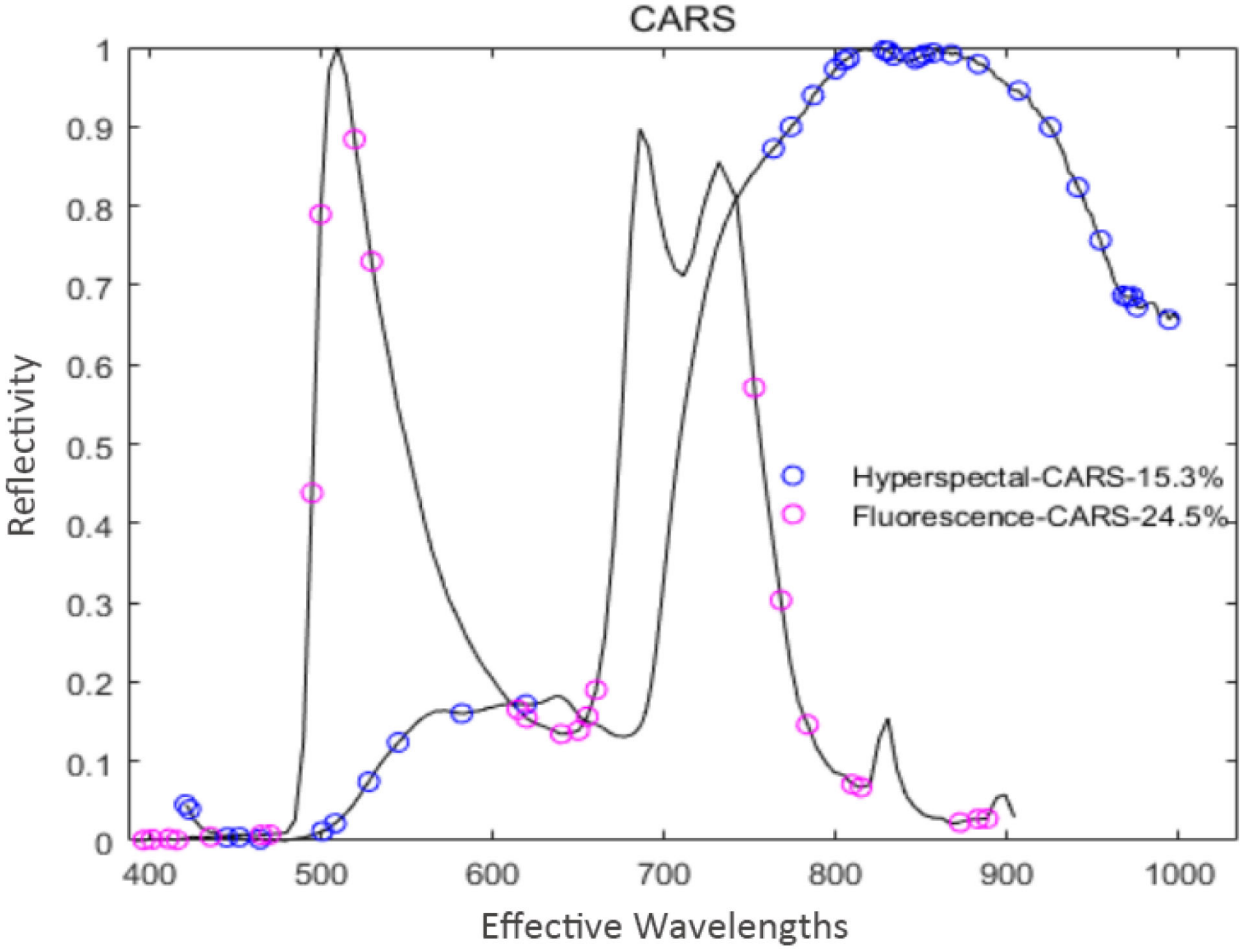
Distribution of the feature variables extracted by CARS.

The curve in **Figure 5C** represents changes in the regression coefficient of each feature variable with the increase of the sampling times. The blue “*” indicates the Monte Carlo sampling times when *RMSECV* had a minimum value. The model prediction performance was optimal at this time, and the corresponding number of samples was 40. Also, the trend of **Figure 6** is similar to **Figure 5**, and the corresponding number of samples was 36 when *RMSECV* had the minimum value. Finally, the numbers of feature variables of hyperspectral data and fluorescence spectral data extracted by CARS were 35 and 25, respectively, accounting for 15.3% and 24.5% of the total original spectral variables. The distribution of feature variables extracted by CARS is shown in **Figure 7**.

As shown in **Figure 7**, the hyperspectral feature variables extracted by CARS were mainly concentrated in two spectral ranges of 430~610 nm and 800~1000 nm. In comparison, the fluorescence spectral feature variables extracted by CARS were mainly concentrated in three spectral ranges of 400~500 nm, 600~680 nm, and 770~900 nm.

#### 3.4.3 Extraction of feature variables based on IVSO

IVSO was used to extract feature variables from the pre-processed spectral data. During the extraction process of hyperspectral data and fluorescence spectral data, the maximum numbers of PC cross-validation were set to 14 and 16, the cross-validation numbers were set to 9 and 7, and the running number of WBMS was set to 1000.

**Figure 8:**
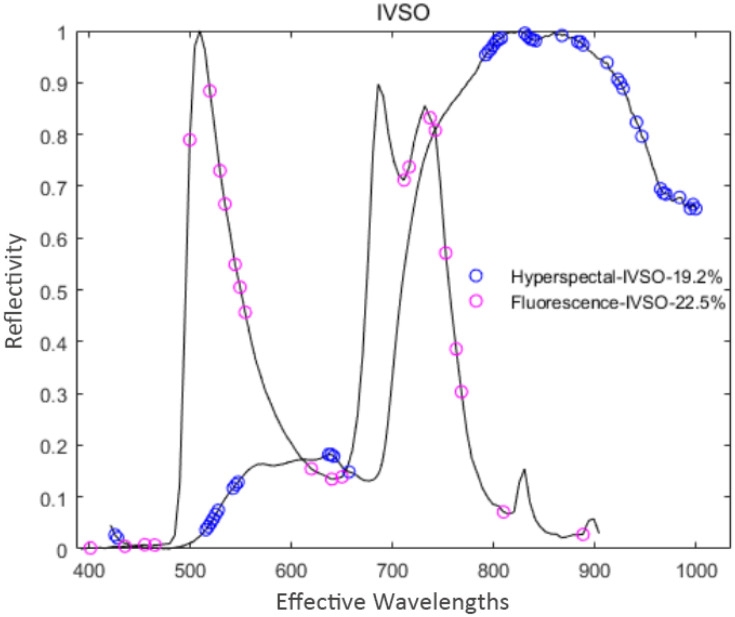
Distribution of the feature variables extracted by IVSO.

In the extraction process of hyperspectral data, IVSO was iterated 9 times. At this time, RMSECV reached a minimum value of 0.807, and 44 feature variables were extracted at the third iteration. In the fluorescence spectral data extraction process, RMSECV reached a minimum value of 0.940, and 23 feature variables were extracted. The distribution of feature variables extracted by IVSO is shown in **Figure 8**, where the hyperspectral feature variables are mainly distributed around 520 nm and 820 nm. In contrast, the distribution of the fluorescence spectral characteristic variables is relatively uniform.

#### 3.4.4 Extraction of feature variables based on IVISSA

**Figure 9:**
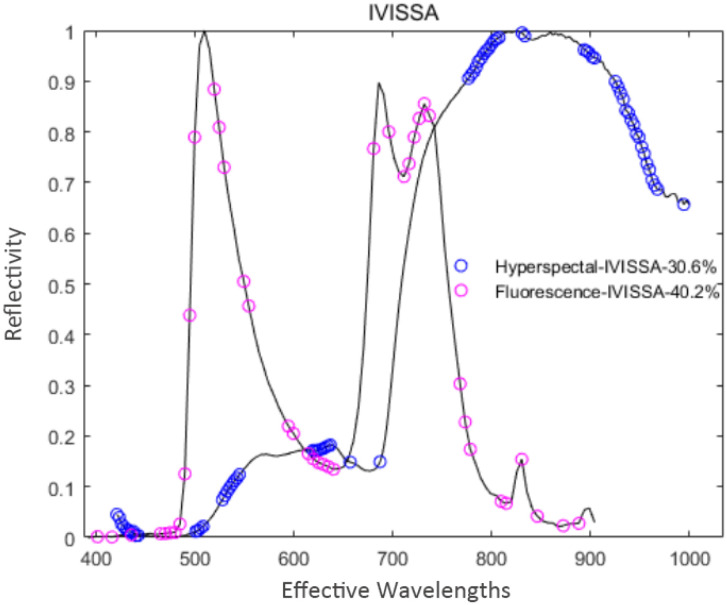
Distribution of feature variables extracted by IVISSA.

IVISSA was used to extract feature variables from the pre-processed spectral data. During the extraction processes of hyperspectral and fluorescence spectral data, the maximum number of latent variables was set to 19 and 17, respectively. Through cross-validation optimization, the number of cross-validation was 10, and the number of binary matrix sampling was 1000. In the extraction process of hyperspectral data, IVISSA iterated a total of 29 times, and *RMSECV* reached a minimum value of 0.7559. At this time, 70 feature variables were extracted, accounting for 30.6% of the total spectral variables. In the fluorescence spectral data extraction process, IVISSA iterated 19 times, and *RMSECV* reached a minimum value of 0.6923. 41 feature variables were extracted at that time, accounting for 40.2% of the total spectral variables. The distribution of feature variables extracted by IVISSA is shown in **Figure 9**.

As shown in **Figure 9**, the numbers of two spectral feature variables extracted by the IVSO algorithm are relatively large, and the number of hyperspectral feature variables is much higher than the fluorescence spectral feature variables. Among them, the fluorescence spectral feature variables are distributed uniformly in the whole spectral range, while the hyperspectral feature variables are densely distributed at 450 nm, 540 nm, 620 nm, 810 nm, and 950 nm.

#### 3.4.5 Extraction of feature variables based on MASS

**Figure 10:**
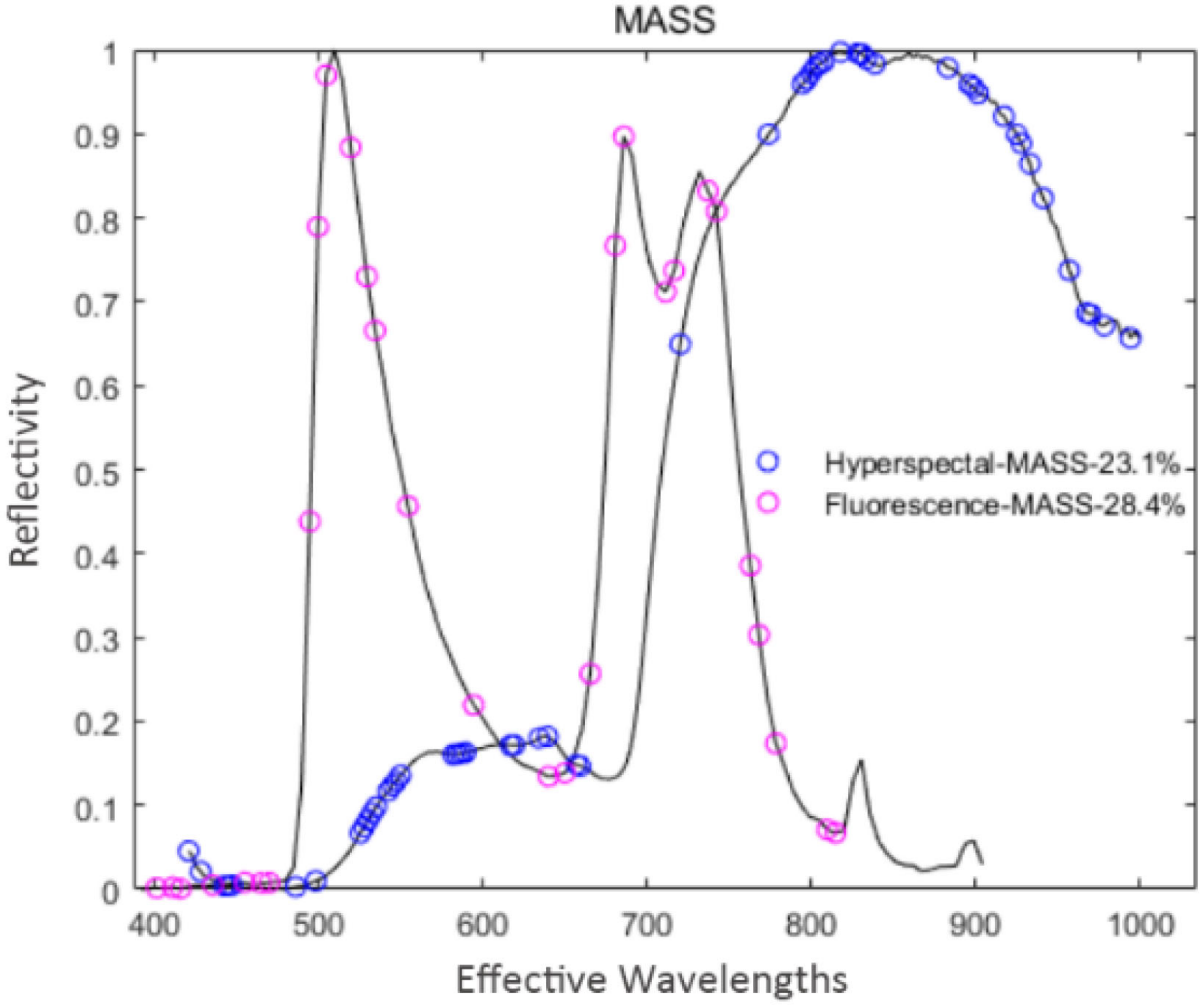
Spectral feature variable distribution map based on MASS.

MASS was used to extract feature variables from the pre-processed spectral data. During the extraction processes of hyperspectral and fluorescence spectral data, the maximum number of latent variables was set to 13 and 14, respectively. Through cross-validation optimization, the number of cross-validation was 5, and the number of binary matrix sampling was 1000. In the extraction process of hyperspectral data, MASS iterated 36 times, and 53 feature variables were extracted, accounting for 23.1% of the total hyperspectral variables. In the extraction process of fluorescence spectral data, MASS iterated 22 times, and 29 feature variables were extracted, accounting for 28.4% of the total fluorescence spectral variables. The distribution of feature variables extracted by MASS is shown in **Figure 10**.

From **Figure 10**, the number of feature variables extracted by MASS for the two types of spectral data are 23.10% and 28.4%, respectively. The extracted fluorescence spectral feature variables are distributed uniformly in the whole range, while the hyperspectral feature variables are concentrated in the former and latter two spectral ranges.

#### 3.4.6 Secondary extraction of the feature variables

The first feature extraction could reduce some redundant and collinear variables in the original feature variables. However, the proportion of first-extracted feature variables is still high, with a few redundant variables. In order to further improve the prediction performance of the model, secondary feature extraction was adopted. Boss could greatly minimize the number of feature variables compared to the other four algorithms. Therefore, combining CARS, MASS, and IVISSA with the Boss algorithm for secondary feature extraction could combine the advantages of different feature extraction algorithms and further reduce the number of feature variables. The number of feature variables after the secondary extraction is listed in **Table 3**.

**Table 3:** The results of secondary feature extraction.

Secondary feature extraction	Hyperspectral		Fluorescence Spectral	
	Number of feature variables	Percentage of total bands	Number of feature variables	Percentage of total bands
CARS-Boss	27	12.1	21	20.2
MASS-Boss	21	9.4	21	20.2
IVISSA-Boss	17	7.59	20	19.6

The specific feature variables obtained by the above three secondary feature extraction methods are listed in **Table 4**.

**Table 4:** Spectral variables obtained by different secondary feature extraction methods.

Feature extraction method	Hyperspectral feature variables	Fluorescence spectral feature variables
MASS-Boss	10,44,45,46,52,53,54,81,88,97,154,155,165, 166,192,201,207,213,217,218,227	3,5,10,14,16,17,22,23,27,29,30,34,42,51,59,60,66,70,75,78,85
CARS-Boss	1,2,11,19,45,52,67,82,139,143,148,153,155, 165,166,172,173,179,185,194,201,207,212, 217,218,219,227	3,5,6,10,16,17,22,23,27,29,46, 47,51,53,54,55,73,76,79,84,99
IVISSA-Boss	1,2,45,52,89,97,147,155,165,166,201, 207,209,212,213,217,227	3,6,10,16,22,23,27,29,33,34,47,50,51,65,66,70,76,78,84,99

#### 3.4.7 Results of feature variable extraction

**Figure 11:**
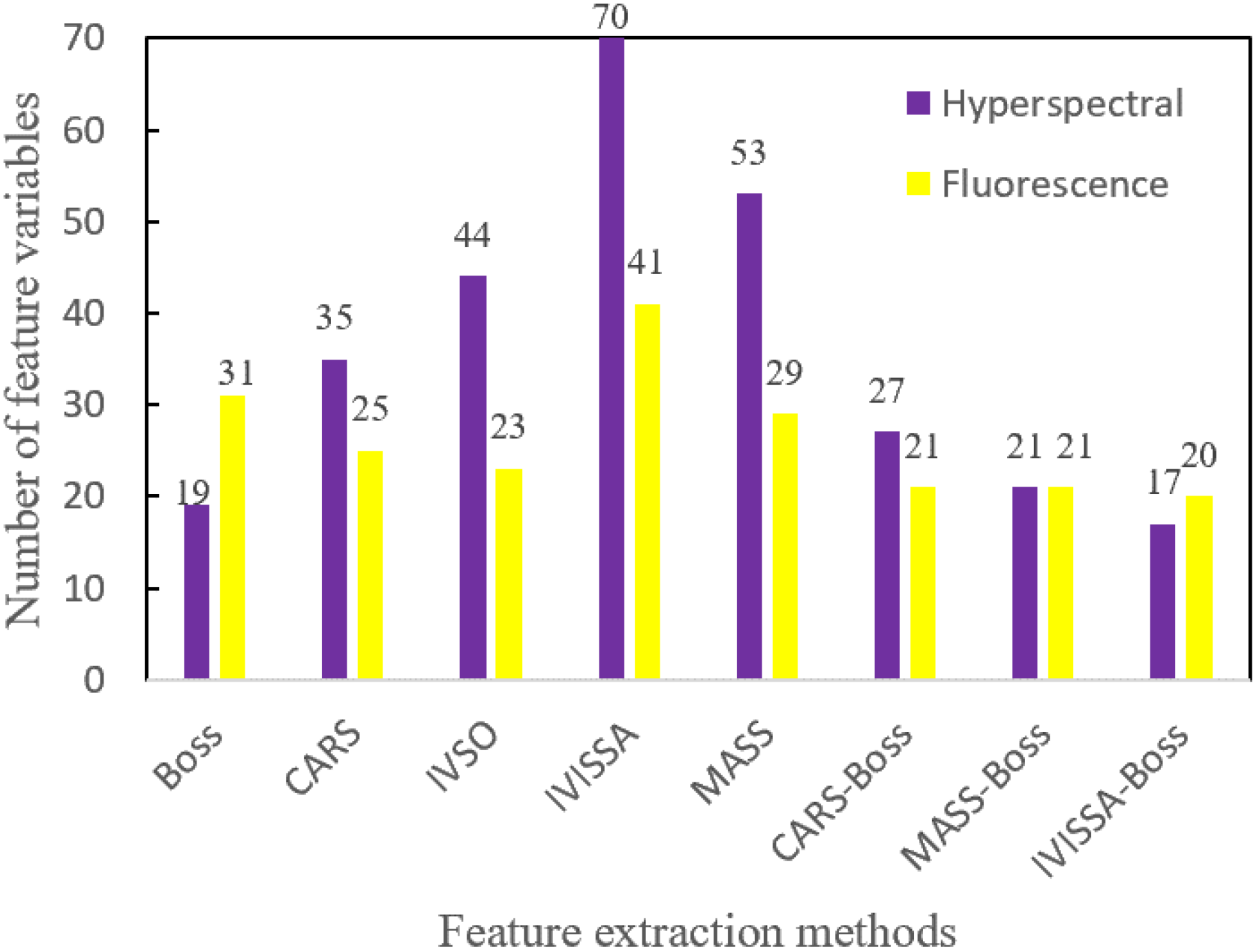
The number of variables extracted by different feature extraction methods.

The numbers of feature variables obtained by the above eight feature extraction methods are shown in **Figure 11**.

From **Figure 11**, for hyperspectral data, the number of extracted feature variables ranged from 17 to 70. Among them, the number of feature variables extracted by IVISSA-Boss is the least, and the number of feature variables extracted by IVISSA is the largest. For the fluorescence spectral data, the number of the extracted feature variables ranged from 20 to 41. Among them, the number of feature variables extracted by IVISSA-Boss is the least, and the number of feature variables extracted by IVISSA is the largest. In addition, the number of feature variables after secondary feature extraction decreased, indicating that secondary feature extraction could further remove the redundant variables.

### 3.5 Performance analysis of predictive models

The extreme learning machine (ELM), the partial least squares regression (PLSR), and the least squares support vector machine optimized by the particle swarm optimization (PSO-LSSVM) prediction models were established for the above-indicated 8 types of feature variables extracted. The differences in the prediction performance of the two spectral image data for the SSC value of kiwifruit were analyzed and compared.

#### 3.5.1 ELM

The “sig” function was selected as the activation function, and the number of neurons in the hidden layer was set from 1 to 100. The prediction results of ELM based on hyperspectral and fluorescence spectral feature variables are listed in **Tables 5**, **6**, respectively.

**Table 5:** Prediction results of ELM based on hyperspectral data.

Feature extraction method	Number of feature variables	Number of hidden neurons	${\text{R}}_{c}^{2}$	*RMSEC*	${\text{R}}_{p}^{2}$	*RMSEP*	*RPD*
Boss	19	24	0.8641	0.5973	0.8372	0.7152	2.48
CARS	35	38	0.9244	0.4747	0.8641	0.5730	2.42
IVSO	44	37	0.9103	0.4507	0.8250	0.8223	2.28
IVISSA	70	36	0.9153	0.4633	0.8064	0.8040	1.90
MASS	53	46	0.9443	0.3795	0.8677	0.6500	2.60
CARS-Boss	27	39	0.9214	0.4188	0.8750	0.6856	2.51
**MASS-Boss**	**21**	**38**	**0.9000**	**0.5433**	**0.8671**	**0.5761**	**2.83**
IVISSA-Boss	17	35	0.9186	0.4626	0.8602	0.6631	2.72

The bold values represent the best performer in each table.

**Table 6:** Prediction results of ELM based on fluorescence spectral data.

Feature extraction method	Number of feature variables	Number of hidden neurons	${\text{R}}_{c}^{2}$	*RMSEC*	${\text{R}}_{p}^{2}$	*RMSEP*	*RPD*
Boss	31	42	0.8805	0.6347	0.8329	0.5434	2.24
CARS	25	50	0.9034	0.5181	0.8784	0.5881	2.73
IVSO	23	47	0.8809	0.5768	0.8530	0.6427	2.45
IVISSA	41	44	0.9004	0.5556	0.8598	0.5275	2.52
MASS	29	55	0.9219	0.4493	0.8889	0.5957	2.88
CARS-Boss	21	54	0.8839	0.5679	0.8689	0.6107	2.62
**MASS-Boss**	**21**	**59**	**0.9429**	**0.4229**	**0.8894**	**0.4824**	**2.88**
IVISSA-Boss	20	65	0.9331	0.4542	0.8755	0.5802	2.67

The bold values represent the best performer in each table.

As shown in **Table 5**, the *RPD* value of ELM established by 8 types of hyperspectral feature variables ranged from 1.90 to 2.83, and its ${\text{R}}_{p}^{2}$ and ${\text{R}}_{c}^{2}$ were higher than 0.86, presenting that the overall prediction effect of ELM is stable. IVISSA-ELM exhibited the worst prediction effect due to more redundant variables in its retained feature variables. The prediction effect of ELM after secondary feature extraction was improved, among which MASS-Boss-ELM showed the best prediction effect with ${\text{R}}_{p}^{2}$ , ${\text{R}}_{c}^{2}$ and RPD of 0.8671, 0.9000, and 2.83, respectively.

**Table 6** illustrates that the RPD value of ELM established by 8 types of fluorescence spectral feature variables ranged from 2.24 to 2.88. Among them, the prediction performance of Boss-ELM is slightly worse, and its RPD is only 2.24. Compared with CARS-ELM, the RPD of CARS-Boss-ELM decreased slightly, and it was estimated that some effective feature variables were excluded in the secondary feature extraction process. The prediction performance of MASS-Boss-ELM was relatively optimal, with ${\text{R}}_{p}^{2}$ , ${\text{R}}_{c}^{2}$ , RPD of 0.8894, 0.9429, and 2.88, respectively.

From **Tables 5**, **6**, the ranges of ${\text{R}}_{p}^{2}$ and ${\text{R}}_{c}^{2}$ of the ELM established by 8 types of hyperspectral feature variables were 0.8064~0.8750 and 0.8641~0.9443, respectively. For the feature variables of fluorescence spectra, the ranges of ${\text{R}}_{p}^{2}$ and ${\text{R}}_{c}^{2}$ corresponding to ELM were 0.8329~0.8894 and 0.8805~0.9429, respectively. Therefore, the overall prediction performance of ELM based on fluorescence spectral data is superior. MASS-Boss-ELM was optimal for both hyperspectral and fluorescence spectral data, verifying that the method reveals the strongest generalization ability.

#### 3.5.2 PLSR

The cross-validation method was used to determine the number of PLSR latent variables, and the optimal latent variables were selected as the final. The prediction results of PLSR based on hyperspectral and fluorescence spectral feature variables are listed in **Tables 7**, **8**, respectively.

**Table 7:** Prediction results of PLSR based on hyperspectral data.

Feature extraction method	Number of feature variables	Number of latent variables (lvs)	${\text{R}}_{c}^{2}$	*RMSEC*	${\text{R}}_{p}^{2}$	*RMSEP*	*RPD*
Boss	19	17	0.8964	0.5214	0.8191	0.7540	2.33
CARS	35	13	0.9179	0.5036	0.7964	0.7013	2.24
IVSO	44	12	0.8709	0.5408	0.8087	0.8598	2.01
IVISSA	70	12	0.8718	0.5698	0.8313	0.7504	2.07
MASS	53	11	0.8505	0.6547	0.8136	0.7041	2.15
CARS-Boss	27	16	0.9081	0.4529	0.8784	0.6762	2.50
**MASS-Boss**	**21**	**17**	**0.8747**	**0.6079**	**0.8717**	**0.5661**	**2.89**
IVISSA-Boss	17	12	0.8727	0.5784	0.8684	0.6433	2.55

The bold values represent the best performer in each table.

**Table 8:** Prediction results of PLSR based on fluorescence spectral data.

Feature extraction method	Number of feature variables	Number of potential variables	${\text{R}}_{c}^{2}$	*RMSEC*	${\text{R}}_{p}^{2}$	*RMSEP*	*RPD*
Boss	31	17	0.8687	0.6612	0.7577	0.6458	2.25
**CARS**	**25**	**15**	**0.8588**	**0.6262**	**0.8159**	**0.7237**	**2.36**
IVSO	23	14	0.8068	0.7344	0.7625	0.8171	2.05
IVISSA	41	30	0.9522	0.3851	0.7467	0.7089	2.20
MASS	29	16	0.8530	0.6164	0.8123	0.7743	2.30
CARS-Boss	21	16	0.8376	0.6715	0.7718	0.8058	2.01
MASS-Boss	21	14	0.8017	0.7880	0.7655	0.7024	1.85
IVISSA-Boss	20	16	0.8438	0.6697	0.7999	0.7293	2.23

The bold values represent the best performer in each table.

From **Table 7**, the PLSR established by 8 types of hyperspectral feature variables performed well in the prediction performance of kiwifruit SSC, and the RPD values exceeded 2.0; the highest RPD value reached 2.89. Compared with PLSR based on first feature extraction, the prediction performance of PLSR after secondary feature extraction was generally improved, indicating that secondary feature extraction could effectively filter out redundant variables. Among them, the prediction results of MASS-Boss-PLSR are relatively the best, with ${\text{R}}_{p}^{2}$ , ${\text{R}}_{c}^{2}$ and RPD of 0.8717, 0.8747 and 2.89, respectively.

As shown in **Table 8**, while comparing with the PLSR after only SG pre-processing, the prediction performance of PLSR displays improvements in the range of 1.85 to 2.36 after both the first and secondary feature variable extraction, both of which are higher than 1.67 of the SG-PLSR without feature extraction (**Table 2**). Among them, the prediction result of MASS-Boss-PLSR is the worst as the secondary feature extraction algorithm eliminates part of the key feature variables. The prediction performance of CARS-PLSR is relatively optimal, with ${\text{R}}_{p}^{2}$ , ${\text{R}}_{c}^{2}$ , and RPD of 0.8159, 0.8588, and 2.36, respectively.

By comparing **Table 7**, **8**, the ranges of ${\text{R}}_{p}^{2}$ and ${\text{R}}_{c}^{2}$ of PLSR based on hyperspectral data are 0.7964~0.8784 and 0.8505~0.9179, respectively. The ranges of ${\text{R}}_{p}^{2}$ and ${\text{R}}_{c}^{2}$ of PLSR based on fluorescence data are 0.7467~0.8159 and 0.8017~0.9522, respectively. By combining **Tables 4**, **7**, there is variability in the performance of the prediction models based on hyperspectral and fluorescence spectral data, in which MASS-Boss-ELM based on fluorescence spectral data is the optimal prediction method, and its ${\text{R}}_{p}^{2}$ ${\text{R}}_{c}^{2}$ , and RPD are 0.8894, 0.9429 and 2.88, respectively.

#### 3.5.3 PSO-LSSVM prediction model

The radial basis function (RBF) was selected as the kernel function of LSSVM, and the prediction performance of the model was easily affected by the regularization parameter γ and the kernel parameter σ^2^ of RBF. The two parameters were optimized by the particle swarm optimization (PSO) algorithm (Bhandari et al., 2015; Bonah et al., 2020). In the training process, the population number, the iteration number, and the initial value of the inertia factor were set to 20,100, and 0.90, respectively, and both the learning factors c_1_ and c_2_ were 2. PSO-LSSVM was tested, and its prediction results are listed in **Tables 9**, **10**, respectively.

**Table 9:** Prediction results of PSO-LSSVM based on hyperspectral data .

Feature extraction method	Number of feature variables	${\text{R}}_{c}^{2}$	*RMSEC*	${\text{R}}_{p}^{2}$	*RMSEP*	*RPD*
Boss	19	0.8403	0.7085	0.8056	0.6227	2.02
CARS	35	0.8126	0.7676	0.7710	0.6758	2.03
**IVSO**	**44**	**0.8231**	**0.7456**	**0.8532**	**0.5411**	**2.54**
**IVISSA**	70	0.8964	0.5710	0.7576	0.6953	1.94
MASS	53	0.8817	0.6098	0.7698	0.6775	1.93
CARS-Boss	27	0.8273	0.7367	0.7805	0.6616	2.10
MASS-Boss	21	0.8265	0.7385	0.8169	0.6042	2.28
IVISSA-Boss	17	0.8651	0.6512	0.7435	0.7152	2.01

The bold values represent the best performer in each table.

**Table 10:** Prediction results of PSO-LSSVM based on fluorescence spectral data.

Feature extraction method	Number of feature variables	${\text{R}}_{c}^{2}$	RMSEC	${\text{R}}_{p}^{2}$	*RMSEP*	*RPD*
Boss	31	0.8987	0.5659	0.6119	0.8922	1.47
CARS	25	0.8739	0.6312	0.7099	0.7713	1.98
IVSO	23	0.8523	0.6832	0.5851	0.9225	1.50
IVISSA	41	0.9582	0.3634	0.7473	0.7199	2.29
MASS	29	0.8677	0.6467	0.7650	0.6942	2.19
CARS-Boss	21	0.8547	0.6777	0.6372	0.8626	1.65
MASS-Boss	21	0.8050	0.7850	0.7206	0.7569	1.61
IVISSA-Boss	20	0.8359	0.7201	0.7691	0.6881	1.97

The bold values represent the best performer in each table.

As exhibited in **Table 9**, the prediction effect of PSO-LSSVM based on 8 types of hyperspectral feature variables performed well, and the *RPD* and ${\text{ R}}_{c}^{2}$ are generally higher than 2.0 and 0.8, respectively, and ${\text{R}}_{p}^{2}$ ranged from 0.74 to 0.85, indicating that PSO-LSSVM presents good prediction performance for the SSC of kiwifruit. Among them, MASS-Boss-PSO-LSSVM illustrates the relatively best prediction results, with ${\text{R}}_{p}^{2}$ , ${\text{R}}_{c}^{2}$ , and RPD of 0.8169, 0.8265, and 2.28, respectively.

The prediction effect of PSO-LSSVM based on fluorescence spectral feature variables is significantly different, and the *RPD* values ranged from 1.47 to 2.29 (**Table 10**). Among them, IVISSA-PSO-LSSVM exhibits the relatively best prediction results, with the ${\text{R}}_{p}^{2}$ , ${\text{R}}_{c}^{2}$ , and RPD of 0.7473, 0.9582 and 2.29, respectively. The prediction performance of PSO-LSSVM is reduced after IVISSA-Boss secondary extraction, indicating that the valid variables among them were over-screened. In addition, the ${\text{R}}_{c}^{2}$ and ${\text{R}}_{p}^{2}$ of all methods differed significantly, specifying that the stability of PSO-LSSVM needs further improvements.

Among these, the best optimization parameters of PSO in the superior predicted models for the two spectra data are listed in **Table 11**.

**Table 11:** The best PSO optimization parameters.

Spectral Type	Feature extraction method	*σ2*	*γ*
Hyperspectral	IVSO	2.3021	3.5083e+06
Fluorescence spectral	IVISSA	3.1623e+08	1.0236e+06

#### 3.5.4 Analysis and comparison of prediction results

**Figure 12:**
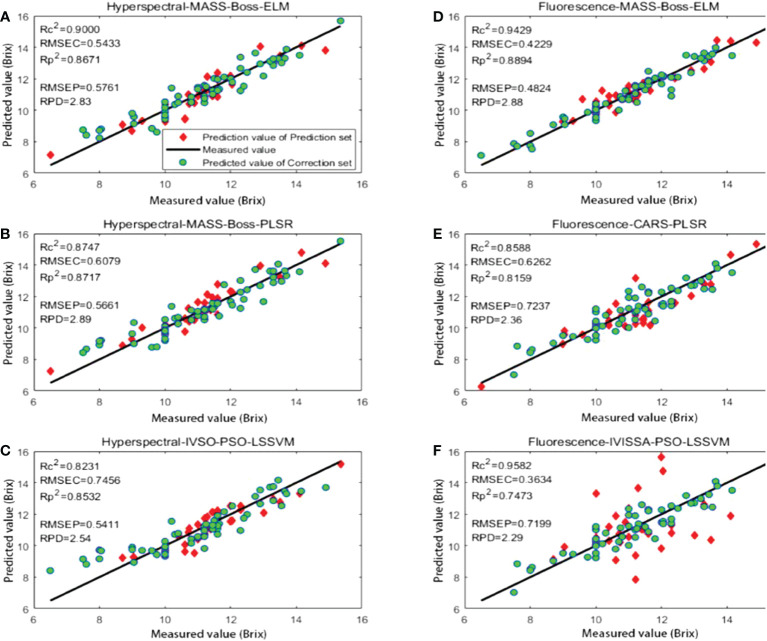
Prediction results of different optimal methods: **(A)** hyperspectral-MASS-Boss-ELM; **(B)** hyperspectral-MASS-Boss-PLSR; **(C)** hyperspectral-IVSO-PSO-LSSVM; **(D)** fluorescence-MASS-Boss-ELM; **(E)** fluorescence-CARS-PLSR; **(F)** fluorescence-IVISSA-PSO-LSSVM.

The methods with relatively superior prediction results based on hyperspectral data and fluorescence spectral data were hyperspectral-BS-MASS-Boss-ELM, fluorescence spectral-SG-MASS-Boss-ELM, hyperspectral-BS-MASS-Boss-PLSR and fluorescence spectral-SG-CARS-PLSR, hyperspectral-BS-MASS-Boss-PSO-LSSVM, and fluorescence spectral-SG-IVISSA-PSO-LSSVM, respectively. The prediction results of the above six methods are shown in **Figure 12** and are listed in **Table 12**.

**Table 12:** Comparison of optimal results based on different feature extraction methods and models.

Spectral Type	Prediction Method	${\text{R}}_{c}^{2}$	*RMSEC*	${\text{R}}_{p}^{2}$	*RMSEP*	*RPD*
Hyperspectral data	MASS-Boss-ELM	0.9000	0.5433	0.8671	0.5761	2.83
	MASS-Boss-PLSR	0.8747	0.6079	0.8717	0.5661	2.89
	IVSO-PSO-LSSVM	0.8231	0.7456	0.8532	0.5411	2.54
Fluorescence spectral data	**MASS-Boss-ELM**	**0.9429**	**0.4229**	**0.8894**	**0.4824**	**2.88**
	CARS-PLSR	0.8588	0.6262	0.8159	0.7237	2.36
	IVISSA-PSO-LSSVM	0.9582	0.3634	0.7473	0.7199	2.29

The bold values represent the best performer in each table.

**Figure 12** shows the regression chart of the prediction results for the above six methods. Comparing the prediction results with the other five methods, the prediction results of IVISSA-PSO-LSSVM based on fluorescence spectral data are quite different, and the prediction results are the worst. This is due to the excessive PSO algorithm parameters and the small number of samples, leading to an overfitting tendency in the training set. Compared with MASS-Boss-PLSR based on hyperspectral data, MASS-Boss-ELM and IVSO-PSO-LSSVM based on hyperspectral data exhibited relatively poor prediction results on the test set. Among them, the predicted results of MASS-Boss-ELM based on fluorescence spectral data illustrated the best generalization ability.

**Table 12** presents that among the prediction results based on hyperspectral data, both MASS-Boss-ELM and MASS-Boss-PLSR show superior prediction performance, indicating that the MASS-Boss secondary extraction method could effectively filter out the feature variables, which could well represent the spectral data. Among them, MASS-Boss-PLSR exhibited a slightly superior ${\text{R}}_{p}^{2}$ RMSEP and RPD to MASS-Boss-ELM could be considered the most suitable prediction method for kiwifruit SSC based on hyperspectral data. The optimal prediction method for kiwifruit SSC based on fluorescence spectral data is MASS-Boss-ELM, whose prediction indicators far exceeded the IVISSA-PSO-LSSVM and CARS-PLSR.

The method followed in this study was compared with those reported in the literature, and the comparison results are listed in **Table 13**. It can be seen from **Table 13** that Moen et al. (Moen et al., 2021) used different machine learning technologies to study the correlation between kiwifruit spectral information and its SSC, and found that the best prediction method was UVE-PLS, with the RMSEP of 1.047 and the ${\text{R}}_{p}^{2}$ of 0.39. Benelli et al. (Benelli et al., 2022) used the PLS model based on hyperspectral imaging technology to evaluate the maturity of “Hayward” kiwifruit, with the ${\text{R}}_{p}^{2}$ was in the range of 0.85~0.94, and *RMSE* was in the range of 1.10-0.73. The best prediction method in this study was MASS-Boss-ELM based on fluorescence spectral data, and its ${\text{R}}_{p}^{2}$ , RMSEP and RPD were 0.8894, 0.4824 and 2.88, respectively. Compared with the previous studies, the ${\text{R}}_{p}^{2}$ obtained in this study has not been improved significantly, but the RMSEP is the lowest, specifying that the MASS-Boss-ELM is superior.

**Table 13:** Comparison of the prediction results with the other methods.

Literature	Method	${\text{R}}_{p}^{2}$	*RMSEP*	*RPD*
Moen, Nilsen, et al., 2021	UVE-PLS	0.3900	1.0470	–
Benelli, Cevoli, et al., 2022	PLS	0.8500~0.9400	1.1000~0.7300	–
This study	MASS-Boss-ELM	0.8894	0.4824	2.88

“-” indicates that RPD was not used in the literature.

## 4 Conclusions

This study explored the efficient prediction of hyperspectral and fluorescence spectral data for nondestructive detection of kiwifruit SSC (soluble solid content). Combining the six pretreatment methods and the PLSR model, the best pre-processing methods for hyperspectral and fluorescence spectral data were BS (boxing smoothing) and SG (Savitzky-Golay), respectively. Then, five primary and three secondary feature extraction algorithms were used to reduce the pre-processed spectral data. Three prediction models have been established: ELM, PLSR, and PSO-LSSVM. The prediction results of PLSR and ELM based on the hyperspectral and fluorescence spectral datasets were better. The best prediction method corresponding to the hyperspectral dataset was MASS-Boss-PLSR, and its ${\text{R}}_{p}^{2}$ , ${\text{R}}_{c}^{2}$ and RPD were 0.8717, 0.8747 and 2.89, respectively. The best prediction method corresponding to the fluorescence spectral dataset was MASS-Boss-ELM, and its ${\text{R}}_{p}^{2}$ , ${\text{R}}_{c}^{2}$ and RPD were 0.8894, 0.9429 and 2.88, respectively. Whereas PSO-LSSVM displayed the worst prediction results. In conclusion, the MASS-Boss-ELM method based on the fluorescence spectral dataset was the best non-destructive prediction method for kiwifruit SSC.

The research methods followed in this study could be improved further. For example, the optimal pre-processing methods for the two types of spectral datasets are different, and the best prediction models for each kind of spectral dataset are also different, which is not conducive to the follow-up research and development of non-destructive testing devices for agricultural products. Therefore, more spectral feature extraction algorithms and different models need to be studied further to find the best prediction model suitable for the different spectral datasets and apply it to the non-destructive testing of other parameters, such as pH and the hardness of kiwifruit.

## Data availability statement

The raw data supporting the conclusions of this article will be made available by the authors, without undue reservation.

## Author contributions

LX: Conceptualization, Data curation, Methodology, Writing – original draft, Writing – review & editing, Investigation, Validation, Formal analysis. YC: Data curation, Methodology, Writing – original draft, Writing – review & editing, Investigation, Validation, Formal analysis. XW: Methodology, Writing – original draft, Writing – review & editing, Investigation, Validation, Formal analysis. HC: Data curation, Formal analysis. ZT: Data curation, Formal analysis. XS: Data curation, Formal analysis. YW: Data curation, Formal analysis, Supervision. ZK: Data curation, Formal analysis. ZZ: Formal analysis. PH: Formal analysis. YH: Supervision, NY: Conceptualization, Supervision, Funding acquisition, Resources. YZ: Conceptualization, Supervision, Funding acquisition, Resources. All authors contributed to the article and approved the submitted version.
